# Comprehensive VR dataset for machine learning: Head- and eye-centred video and positional data

**DOI:** 10.1016/j.dib.2024.111187

**Published:** 2024-11-29

**Authors:** Alexander Kreß, Markus Lappe, Frank Bremmer

**Affiliations:** a Department of Neurophysics, Philipps University Marburg, Karl-von-Frisch Straße 8a, 35043 Marburg, Hesse, Germany; b Institute of Psychology, University Münster, Fliednerstraße 21, 48149 Münster, North Rhine-Westphalia, Germany

**Keywords:** Eye tracking, Head tracking, Deep learning, Spatial navigation, Foraging behaviour, Behavioural data, Naturalistic VR locomotion

## Abstract

We present a comprehensive dataset comprising head- and eye-centred video recordings from human participants performing a search task in a variety of Virtual Reality (VR) environments. Using a VR motion platform, participants navigated these environments freely while their eye movements and positional data were captured and stored in CSV format. The dataset spans six distinct environments, including one specifically for calibrating the motion platform, and provides a cumulative playtime of over 10 h for both head- and eye-centred perspectives.

The data collection was conducted in naturalistic VR settings, where participants collected virtual coins scattered across diverse landscapes such as grassy fields, dense forests, and an abandoned urban area, each characterized by unique ecological features. This structured and detailed dataset offers substantial reuse potential, particularly for machine learning applications.

The richness of the dataset makes it an ideal resource for training models on various tasks, including the prediction and analysis of visual search behaviour, eye movement and navigation strategies within VR environments. Researchers can leverage this extensive dataset to develop and refine algorithms requiring comprehensive and annotated video and positional data. By providing a well-organized and detailed dataset, it serves as an invaluable resource for advancing machine learning research in VR and fostering the development of innovative VR technologies.

Specifications Table

**Table:** 

Subject	Neuroscience.
Specific subject area	Self-motion, navigation and visual search in Virtual Reality (VR)
Type of data	Table, Video, Raw, Processed.
Data collection	For data collection participants were tasked to collect coins in different virtual environments. Movement within the virtual reality was realized by the participant walking on a VR motion platform (Virtualiser Elite 2 by Cyberith). During the experiment the participants were wearing an HTC Vive Pro Eye VR Headset with a build in Tobii Eye tracker (120 Hz sampling rate). During the experiment head position and rotation, hip position and rotation as well as gaze position were recorded.
Data source location	• Institution: The Adaptive Mind – TAM DataHub • City/Town/Region: Marburg • Country: Germany.
Data accessibility	Repository name: Comprehensive VR Dataset for Machine Learning: Head- and Eye-Centred Video and Positional Data Data identification number: 10.60834/tam-datahub-6.2 Direct URL to data: 10.60834/tam-datahub-6.2 Instructions for accessing these data: Download the desired archive files and unpack all of them into the same directory.
Related research article	none.

## 1 Value of the Data

• This dataset is valuable for studying scenery-dependent self-motion and navigational behaviours and visual search in quasi natural settings.
• It is also useful for training computer vision models and computational models of visual search, as it includes annotated gaze data along with head and eye-centered video recordings.
• Researchers can leverage this data to improve algorithms for gaze estimation, gaze prediction and enhance the accuracy of head movement tracking.
• The inclusion of annotated gaze positions allows for detailed analysis and validation of theoretical models in neuroscience.
• This data can also be applied to develop and refine augmented reality and virtual reality applications by understanding user gaze and head movement patterns.

## 2 Background

The data was collected to train a Variational Autoencoder with the purpose of parameterizing visual self-motion stimuli as well as predict heading and gaze parameters from a given set of parameters. In addition, the data was also used to train a modified 3D ResNet to predict heading and gaze parameters in a goal driven approach to model parts of the primate dorsal visual pathway in a similar approach to DorsalNet [1].

## 3 Data Description

The dataset comprises head- and eye-centered videos recorded from human participants performing a search task in a Virtual Reality (VR) environment. In blocks of trials, participants navigated freely using a VR motion platform to enable a naturalistic mode of locomotion. Eye and positional data were recorded and saved in CSV format, corresponding to the recorded video files.

**Fig. 1:**
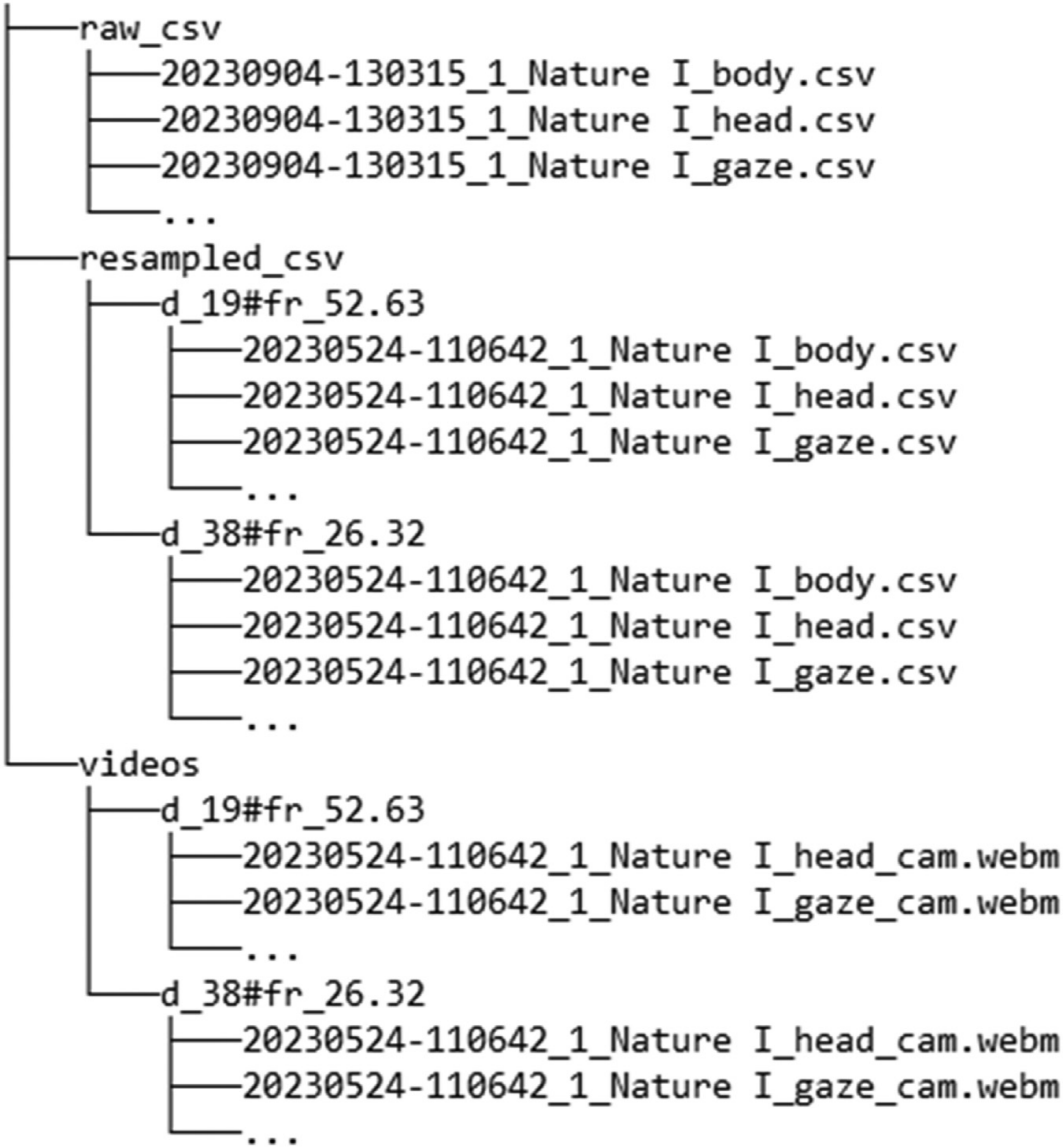
Structure of the dataset. Raw csv, resampled csv and video files each have their respective directory. Resampled data is further subdivided into 52.63 Hz and 26.32 Hz sampling rate.

The dataset is organized into three main directories as illustrated in Fig. 1.

1. raw_csv: Contains the original body, head, and gaze data as recorded during the experiment.
2. resampled_csv: Includes data resampled to 26.32 Hz and 52.63 Hz.
3. videos: Contains video files recorded from the replay of the resampled data.

Each CSV file is structured identically, with separate files for body, head, and gaze data. The filenames include the recording start date and time, recording block number and environment name. Table 1 details the CSV column descriptions. Note that all data is recorded in global coordinates, with pitch and yaw of gaze given in eye-in-head coordinates. Since Unity was used for the creation of the different VR environments, presentation, and data recording, the y-axis denotes the upwards direction.

**Table 1:** Description of the columns in the csv data. Note, that all data is recorded in global coordinates, while the pitch and yaw of the gaze is given in eye in head coordinates.

Column	Description	UNIT
TIME	Time in seconds since start of recording	s
POS.X	X position relative to ground plane	Degree [°]
POS.Y	Y position relative to ground plane (up)	Degree [°]
POS.Z	Z position relative to ground plane	Degree [°]
ROT.X	Pitch	Degree [°]
ROT.Y	Yaw	Degree [°]
ROT.Z	Roll	Degree [°]

The dataset includes annotated video data from six environments, one of which was used solely for motion platform calibration. The total playtime across all environments is 10:21:50 h for both head- and eye-centered data. Table 2 provides the cumulative playtime as well as a short description of the scenery for each environment. The environments where designed to simulate a diverse collection of natural sceneries, including grassy fields, dense forests, and an abandoned urban area.

**Table 2:** Cumulative video playtime for each environment. The given playtime is equal for head- and eye-centred videos. A short description of each scenery is given in the last column.

Environment	Sample image	Cumulative play time	Description
ENDLESS HALLWAY		00:02:06	A straight corridor with striped walls, that infinitely repeats itself.
NATURE I		02:16:22	A serene and open grassy field environment with sparse trees and bushes.
NATURE II		06:13:29	An expanded and denser version of Nature I, featuring additional trees, vegetation and other points of interest.
HDR FORREST		00:36:44	A darker, more realistic forest environment with less vegetation on eye level.
FLODDED GROUNDS		01:02:29	An urban environment characterized by waterlogged areas, featuring flooded fields and marshlands as well as a selection of buildings with an explorable interior.
FANTASY FORREST		00:10:37	A dense forest featuring small, steep hills and vibrant colours

**Fig. 2:**
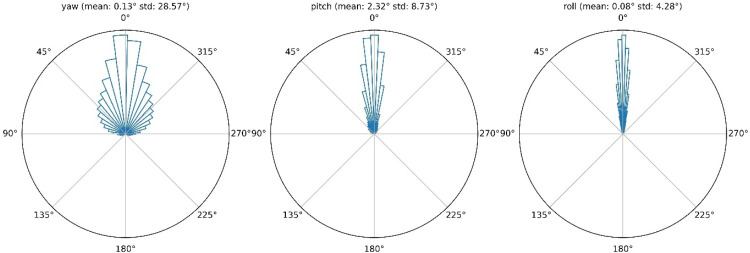
Distribution of heading direction collapsed across environments. Shown, are the yaw, pitch and roll of the head relative to the current heading direction. Mean and standard deviation of the distributions are given in the heading of the respective subplot.

Heading direction was exclusively forward, as the participants did at no point walk backwards. The distribution of heading directions collapsed across environments can be seen in Fig. 2.

## 4 Experimental Design, Materials and Methods

### 4.1 Participants

The study sample comprised 14 participants, consisting of seven males and six females, all recruited from a university student population. Consequently, the cohort consisted entirely of young adults, with ages ranging from 20 to 30 years. All participants possessed normal or corrected-to-normal vision. Individuals requiring corrective lenses were able to use their glasses in conjunction with the virtual reality (VR) headset, ensuring consistent visual acuity across participants.

### 4.2 Experimental design and data collection

The data collection for this study was designed to simulate naturalistic environments within a Virtual Reality (VR) framework. The creation and presentation of all scenes to the participants were performed using the Unity game engine (version 2021.3.1f1 [2]), with freely available assets for the maps sourced from the Unity Asset Store [3,4]. Participants navigated these environments using a VR motion platform [5], with their primary task being the collection of virtual coins dispersed throughout varied landscapes.

Participants were immersed in five distinct environments, each crafted to present unique ecological features: (1) a small grassy field adorned with sparse trees and bushes; (2) an expanded version of the grassy field featuring denser tree populations and notable points of interest; (3) a darker, more lifelike forest; (4) a densely vegetated, hilly forest; and (5) an abandoned urban area with accessible buildings. Additionally, a straight corridor was utilized for system testing and calibration purposes.

In each environment, coins were strategically placed at diverse locations. The number of coins varied between six and eleven per environment, all of which were instantiated at the beginning of each trial, allowing participants to collect them in any order. To mitigate motion sickness, participants were given the flexibility to take breaks as needed.

Prior to each trial, a thorough calibration process was conducted. The calibration involved a two-step procedure: First, the eye tracker was calibrated using the HTC Vive Pro Eye Headsetʼs built-in 5-point calibration system [6]. Second, the forward direction of the motion platform was aligned. This was achieved by instructing the participants to stand upright and look into the direction their hips were facing, which was then synchronized with their head orientation in Unity, ensuring precise directional control.

Post-calibration, the motion platform enabled highly accurate and naturalistic 360° movement. Throughout each trial, positional and rotational data were recorded and stored in CSV format. The recorded parameters included body position in world space, head position in world space, and gaze direction relative to the head orientation.

Following the recording sessions, the collected data underwent resampling at frequencies of 26.32 Hz and 52.63 Hz. The resampled data were re-imported into Unity, where the participants’ movements were replayed for further analysis. During this replay, both head and gaze camera videos were recorded, with the output saved as uncompressed WebM files. The head camera was positioned centrally within the head, aligned with the participant's head orientation, while the gaze camera was co-located but adjusted according to the instantaneous gaze direction.

### 4.3 VR motion platform

Due to the significant impact of the gait cycle on head position, a VR motion platform allowing unrestricted vertical hip movement was utilized, enabling participants to move freely within the virtual environment. Specifically, we used the Virtualizer ELITE 2 © [5], an advanced omni-directional VR treadmill designed to enhance immersive virtual reality experiences. It features an actively powered motion platform that supports user gait through dynamic adjustments, significantly reducing physical effort and enabling to capture the influence gait has on head position and retinal optic flow. The system is equipped with six optical motion sensors operating at a sample rate of 1000 Hz which are located in the base of the platform, that track the speed and direction of the feet. In addition, an optical rotation sensor for precise hip orientation tracking and an optical height sensor to monitor user hip height are used in the device to accurately define the forward direction of motion within the virtual environment. These sensors enable detailed and accurate capture of the participants’ movement direction, speed, and user orientation, supporting a variety of actions such as walking, running, crouching, and backward movements.

### 4.4 HTC Vive Pro Eye VR headset

The HTC Vive Pro Eye VR headset [7] was employed for its advanced eye-tracking capabilities, providing detailed and accurate data essential for studying user interactions within virtual environments. This headset features dual OLED displays with a combined resolution of 2880 × 1600 pixels (1440 × 1600 pixels per eye) and a 90 Hz refresh rate, delivering a 110-degree field of view. The integrated Tobii eye-tracking technology operates at a frequency of 120 Hz, with an accuracy between 0.5° and 1.1° in the center 20° field of view, and includes a 5-point calibration system. The eye tracker captures gaze origin, gaze direction, pupil position, pupil size, and eye openness, facilitating precise user attention mapping. However, for this dataset only gaze direction was recorded.

## Limitations

Not applicable.

## Ethics Statement

This research involved human participants and was conducted in full accordance with the principles outlined in the Declaration of Helsinki. Informed consent was obtained from all participants prior to their inclusion in the study. Consistent with the requirements of the Deutsche Forschungsgemeinschaft (DFG) [8], the study design incorporated a research ethics self-assessment [9], thereby obviating the need for formal ethical committee approval.

## CRediT Author Statement

**Alexander Kreß:** Conceptualization, Methodology, Software, Writing - Original Draft, Visualization, **Frank Bremmer:** Supervision, Funding acquisition, Conceptualization, Writing - Review & Editing. **Markus Lappe:** Supervision, Conceptualization, Writing - Review & Editing.

## Data Availability

The Adaptive Mind DatahubComprehensive VR Dataset for Machine Learning: Head- and Eye-Centred Video and Positional Data (Original data).
